# Role of FK506 binding protein 51 in central nervous system diseases

**DOI:** 10.3389/fnmol.2025.1725945

**Published:** 2026-01-12

**Authors:** Haokun Peng, Yanhao Wei, Yanmei Qiu, Rentang Bi, Longhai Zeng, Bo Hu, Yanan Li

**Affiliations:** Wuhan Union Hospital, Tongji Medical College, Huazhong University of Science and Technology, Wuhan, China

**Keywords:** FKBP51, CNS diseases, tauopathy, neuroinflammation, SAFit

## Abstract

FK506-binding protein 51 (FKBP51) is a pivotal molecular chaperone and scaffolding protein that integrates and modulates multiple signaling pathways—including those involving HSP90, the glucocorticoid receptor, AKT, and NF-κB—through its FK1, FK2, and TPR domains, thereby playing a central role in the maintenance of central nervous system (CNS) homeostasis. This review systematically elaborates on the pathological mechanisms and therapeutic potential of FKBP51 in a variety of CNS disorders. In neurodegenerative diseases, FKBP51 promotes aberrant aggregation of Tau protein via the HSP90 complex, exacerbating the pathological progression of Alzheimer’s disease; in Parkinson’s disease, it influences neuronal survival through interaction with the PINK1/AKT signaling pathway; while in Huntington’s disease, it impairs the clearance of mutant huntingtin (mHTT) protein. In models of ischemic stroke, upregulation of FKBP51 enhances autophagy and inflammatory responses through pathways such as AKT/FoxO3, thereby amplifying brain injury. In glioma, FKBP51 exhibits a context-dependent dual role: it may exert tumor-suppressive effects by inhibiting Akt, while its splice variant FKBP51s can regulate PD-L1 expression, promoting tumor immune evasion and therapy resistance. Emerging highly selective small-molecule inhibitors, gene-editing technologies, and novel applications of conventional drugs targeting FKBP51 have demonstrated significant interventional potential in preclinical studies. In summary, FKBP51 constitutes a pleiotropic signaling node, positioning it as a prime therapeutic target for a broad spectrum of CNS disorders.

## 1 Introduction

FK506 binding protein 51 (FKBP51), first identified in 1990 (Smith et al., 1990), is an immunophilin that has garnered considerable attention for its multifaceted roles in physiology and disease. As a member of the chaperone protein family with peptidyl-prolyl cis-trans isomerase (PPIase) activity, FKBP51 is widely distributed across various tissues (Annett et al., 2020). Within the central nervous system (CNS), FKBP51 is abundantly expressed in regions critical for learning, memory, emotion, and stress regulation, such as the hippocampus, hypothalamus, amygdala, and cerebral cortex, underscoring its importance in neural function.

For a long time, research on FKBP51 has primarily focused on its implications in psychiatric disorders. As a critical negative regulator of the glucocorticoid receptor signaling pathway, FKBP51 plays an important role in the stress response mediated by the hypothalamic–pituitary–adrenal (HPA) axis (Fries et al., 2017). Extensive genetic and epigenetic studies have demonstrated that polymorphisms in the FKBP51 gene and alterations in its expression levels are closely associated with the risk and treatment response of several psychiatric conditions, such as major depressive disorder, post-traumatic stress disorder, and anxiety disorders, thereby establishing its importance in psychopathology (Criado-Marrero et al., 2018; Matosin et al., 2018; Zannas et al., 2016).

However, recent investigations have substantially broadened the functional scope of FKBP51. Accumulating evidence now implicates FKBP51 in a wide spectrum of neurological disorders, far beyond its established roles in stress and psychiatry. FKBP51 dysregulation has been documented across a spectrum of neurological disorders—encompassing neurodegenerative pathologies such as Alzheimer’s, Parkinson’s, and Huntington’s diseases, along with cerebrovascular events and gliomas—where it actively participates in disease mechanisms (Chakraborty and Zweckstetter, 2025; Garcia-Gomara et al., 2025; Koren et al., 2011; Liu et al., 2022; Sarkar et al., 2008; Yang et al., 2015). These emerging insights underscore FKBP51’s engagement in fundamental neuropathological processes, including tau protein hyperphosphorylation, α-synuclein-mediated toxicity, neuroinflammatory cascades, and autophagic dysregulation.

This review systematically synthesizes the fundamental characteristics and recent advances concerning FKBP51’s functions across neurological disorders. We particularly focus on elucidating its complex, and sometimes dual, roles in pathogenesis. By integrating these multifaceted insights, we aim to provide a cohesive conceptual framework that not only clarifies FKBP51’s neurobiological mechanisms but also highlights its promising potential as a therapeutic target for various neurological conditions.

## 2 Structure of FKBP51: FK1, FK2, TPR

**FIGURE 1:**
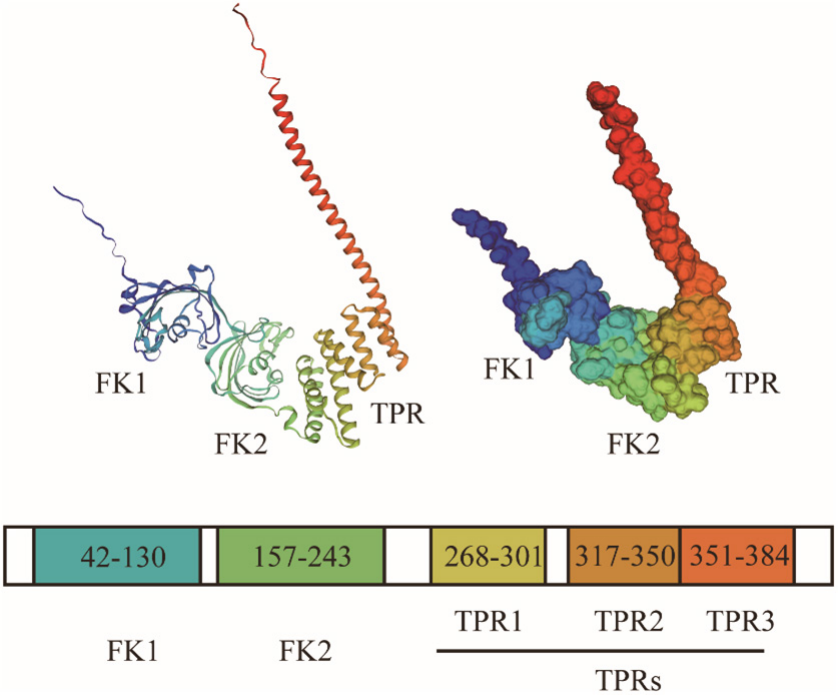
Structural domain composition and three-dimensional crystal structure of FKBP51 (PDB-ID:1KT0). FK1 is located at the N-terminus, surrounded by five antiparallel β-strands that bend around a central α-helix, forming a conical shape with a hydrophobic groove. The hydrophobic groove of the FK1 domain constitutes the primary binding pocket for immunosuppressants like FK506 and is essential for its PPIase activity. FK2 presents a structure similar to FK1. The TPR domain is located at the C-terminus of FKBP51, consisting of three TPR motifs and an additional helix, which fold into 7 antiparallel α-helices. The amphipathic groove of the TPR domain is precisely configured to recognize and bind the C-terminal MEEVD motif of HSP90, a interaction critical for FKBP51’s incorporation into chaperone complexes.

FKBP51 is a 51-kDa protein characterized by three functional domains: two FK506-binding domains (FK1 and FK2) at the N-terminus and a tetratricopeptide repeat (TPR) domain at the C-terminus, as elucidated by X-ray crystallography (Sinars et al., 2003; Figure 1).

The FK1 domain, located at the N-terminus, harbors PPIase activity and represents a unique structural feature within the FKBP family. Its conical shape, formed by five antiparallel β-strands encircling a central α-helix, features a hydrophobic groove crucial for substrate binding (Barik, 2006). As a PPIase, FK1 catalyzes the cis-trans isomerization of peptidyl-prolyl bonds within client proteins, facilitating conformational changes that are essential for protein folding, transport, and cell cycle regulation (Ghartey-Kwansah et al., 2018; Göthel and Marahiel, 1999; Laplante and Sabatini, 2012). Importantly, FKBP51’s cellular functions are not solely dependent on its enzymatic activity, rather it also relies on its domains as platforms for protein interaction and regulation (Soto et al., 2023).

The FK2 domain, while structurally homologous to FK1, has evolved a primary role in protein-protein scaffolding. Although it retains residual PPIase activity, its catalytic efficiency is markedly lower than that of FK1, suggesting that its scaffold function is of greater physiological importance (Zgajnar et al., 2019). This domain primarily facilitates high-affinity protein-protein interactions, as evidenced by the critical role of residues D195, H196, and D197 within FK2: their deletion ablates binding to the progesterone receptor (PR) complex (Sinars et al., 2003).

The C-terminal TPR domain of FKBP51 acts as a critical scaffold for protein-protein interactions and serves as a binding site for various heat shock proteins and tubulin. Comprising three tandem TPR motifs (each ∼34 residues) and an additional helix, it folds into seven antiparallel α-helices that form an amphipathic groove for target peptide recognition (D’Andrea and Regan, 2003). FKBP51 specifically binds the HSP90 C-terminal MEEVD motif via this TPR groove, utilizing electrostatic interactions with the C-terminal DV residues and hydrophobic contacts with the MEEV segment (Cheung-Flynn et al., 2003). Notably, multiple TPR-domain-containing proteins compete for binding to the HSP90 MEEVD motif, with specificity determined by differences in binding affinity (Hähle et al., 2019). While the TPR domain mediates the primary interaction with HSP90, recent studies indicate that the FK1 and FK2 domains also contribute to the overall stability of the FKBP51-HSP90 complex (Oroz et al., 2018).

## 3 Signaling pathways regulated by FKBP51

**FIGURE 2:**
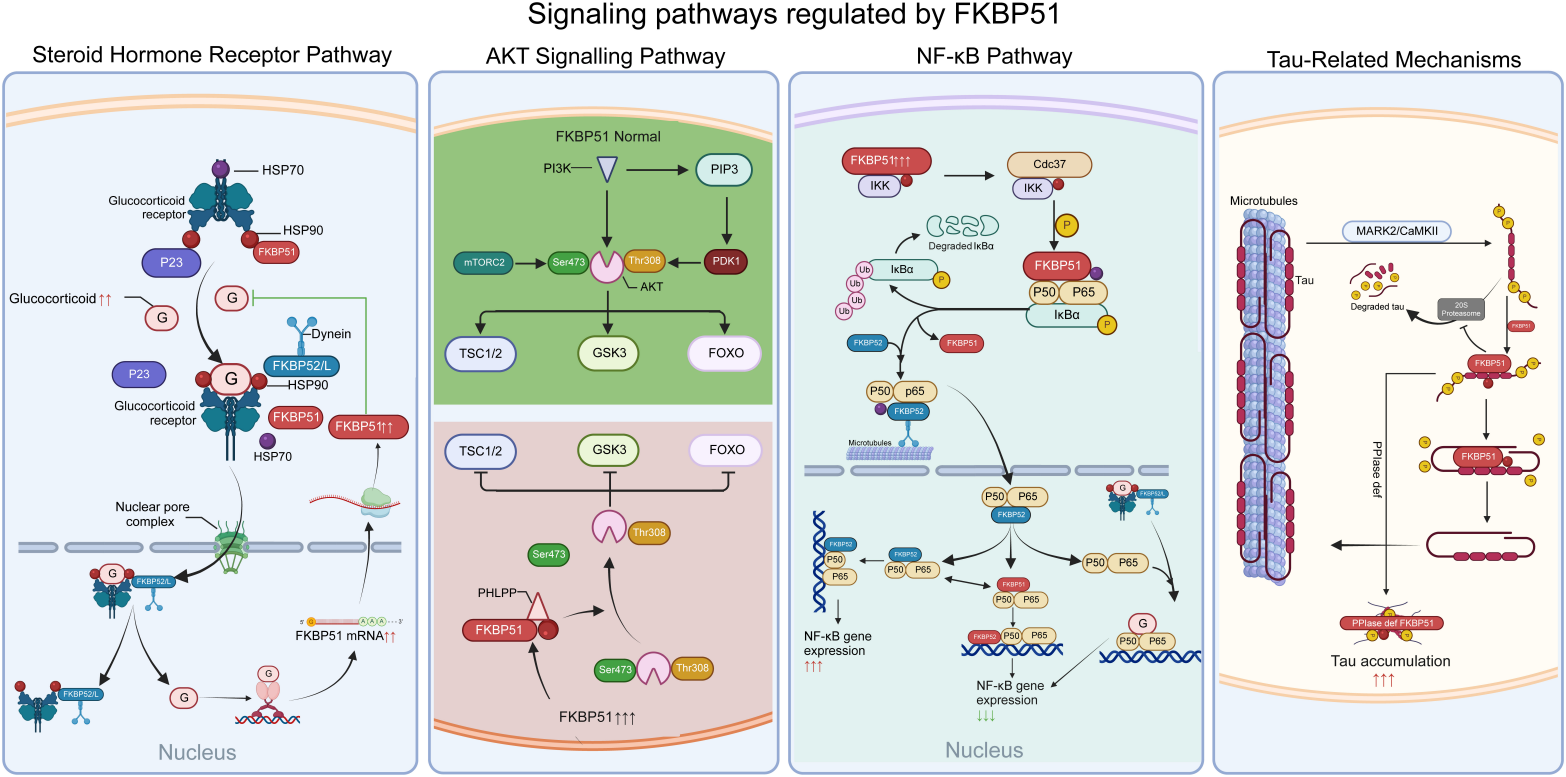
Summary of FKBP51 mechanisms, including steroid receptor pathways, PHLPP/AKT pathway, NF-κB pathway, and tau protein. (1) Glucocorticoid receptor pathway. GR forms a mature complex with HSP90 dimer and p23. FKBP51 competitively displaces FKBP52, blocking dynein-dynactin motor recruitment. FKBP51 binding induces conformational changes in GR that mask nuclear localization signals (NLS), slowing microtubule-dependent transport. (2) PHLPP/AKT pathway. FKBP51, PHLPP and AKT form a complex, which facilitates the dephosphorylation the hydrophobic motif (Ser473 in AKT) of AKT, thus negatively regulating AKT activity. (3) NF-κB pathway. FKBP51 binds with all three subunits of IKK, IKKα, IKKβ, and IKKγ, providing its scaffold function. Additionally, FKBP51 also acts as an isomerase, inhibiting IKK’s kinase activity. (4) tau protein. FKBP51 catalyzes the isomerization of p-Tau protein from the trans to the cis conformation at proline heterogeneity.

FKBP51 functions as a key regulatory node by integrating several critical signaling pathways, as summarized in Figure 2. These include steroid hormone receptor signaling, the AKT pathway, the NF-κB pathway, and tau protein homeostasis (Figure 2).

### 3.1 Steroid hormone receptor pathway

Initially identified within steroid receptor complexes, FKBP51 has been extensively studied for its interaction with steroid hormone receptors (SHRs) (Smith et al., 1990). SHRs are nuclear receptor transcription factors regulating critical physiological processes such as development, reproduction, and metabolism (Cole et al., 2019; Muldoon, 1980; Weikum et al., 2018). These include the glucocorticoid receptor (GR), mineralocorticoid receptor (MR), estrogen receptor (ER), androgen receptor (AR), and progesterone receptor (PR). FKBP51 is widely recognized as a negative regulator of GR, MR, and PR activity (Fries et al., 2017; Gallo et al., 2007; Hubler et al., 2003; Reynolds et al., 1999).

As a co-chaperone, FKBP51 assembles into multimeric complexes with SHRs, molecular chaperones, and other co-factors to facilitate hormone binding and nuclear translocation. Mature SHRs form heterocomplexes with an HSP90 dimer, HSP70, the co-chaperone p23, and a single TPR-domain immunophilin (Noddings et al., 2023; Schopf et al., 2017; Wang et al., 2022; Zgajnar et al., 2019). For example, in the GR complex, ligand-free GR initially associates with an HSP90 dimer and p23 to form a maturation-competent heterocomplex. Upon glucocorticoid binding, FKBP51 competitively displaces FKBP52 from the GR-HSP90 complex. This displacement prevents recruitment of the dynein-dynactin motor complex, thereby delaying microtubule-dependent nuclear translocation by 30%–50% compared to FKBP52-bound complexes (Weikum et al., 2018). Structural studies reveal that FKBP51 binding induces conformational changes in GR, partially occluding nuclear localization signals (NLS) and reducing importin-α affinity (Noddings et al., 2023). Notably, glucocorticoids strongly induce FKBP51 expression at both mRNA and protein levels (Baughman et al., 1997; Vermeer et al., 2003), establishing an ultrashort negative feedback loop that limits GR activity.

### 3.2 AKT signaling pathway

AKT, also known as Protein Kinase B (PKB), is a serine/threonine kinase central to regulating cell survival and apoptosis. AKT dysregulation is implicated in a wide range of diseases, including cancer, cardiovascular diseases, insulin resistance, type 2 diabetes, inflammation, autoimmune disorders, and neurological disorders (Jones et al., 1991; Manning and Toker, 2017). Increasing evidence points to a significant interaction between FKBP51 and the AKT-signaling pathway.

Pei et al. (2009) reported that FKBP51 knockdown in cancer cells increases AKT phosphorylation, enhancing resistance to genotoxic stress and decreasing apoptosis. Conversely, FKBP51 overexpression significantly reduces the AKT phosphorylation and its downstream targets, such as glycogen synthase kinase-3β (GSK-3β) and forkhead box O1 (FOXO1). Biochemical studies demonstrate that FKBP51 scaffolds the formation of a ternary complex with both AKT and PH domain leucine-rich repeat protein phosphatase (PHLPP) (Pei et al., 2009). Specifically, FKBP51 recruits PHLPP via its TPR domain while simultaneously binding AKT through FK1/FK2 domains, thereby positioning PHLPP to dephosphorylate AKT at Ser473 (hydrophobic motif) — a critical step for AKT inactivation (Gao et al., 2005; Manning and Toker, 2017; Revathidevi and Munirajan, 2019). In addition to directly scaffolding AKT and PHLPP, FKBP51 may also influence AKT signaling through its interaction with HSP90. FKBP51 inhibitor, small molecule inhibitor (SAFit) inhibits AKT signaling through increasing the phosphorylation of AS160 (Balsevich et al., 2017). Acting as a scaffold protein, FKBP51 facilitates the juxtaposition of PHLPP and AKT, promoting AKT dephosphorylation and subsequently regulating AKT activity (Pei et al., 2009; Wang, 2011).

### 3.3 NF-κB pathway

NF-κB is a central transcription factor that regulates inflammation, cellular growth, maturation, differentiation, and apoptosis (Capece et al., 2022; Hayden and Ghosh, 2011; Hayden et al., 2006; Zhang et al., 2017). FKBP51 positively influences NF-κB activity. Genetic knockdown or overexpression of FKBP51 significantly modulates NF-κB activity (Jiang et al., 2008; Komura et al., 2005; Romano et al., 2010, 2015; Srivastava et al., 2015). The mechanism by which FKBP51 interfaces with the NF-κB signaling cascade has been elucidated. FKBP51 participates in the NF-κB signaling pathway primarily by scaffolding the IKK complex and modulating its activity through peptidyl-prolyl isomerization (Jiang et al., 2008; Romano et al., 2015).

In its inactive state, the NF-κB dimer is bound by inhibitor IκB, preventing nuclear translocation. Upon activation, the IKK complex phosphorylates IκB, freeing NF-κB, freeing NF-κB to enter the nucleus and activate gene transcription (Capece et al., 2022; Hayden and Ghosh, 2011). In melanoma cells, FKBP51 knockdown inhibited doxorubicin-induced NF-κB activation and nuclear translocation by disrupting IKK-mediated phosphorylation of IκB (Romano et al., 2004). Romano et al. (2015) further demonstrated that FKBP51 interacts with all subunits of the IKK complex (IKKα, IKKβ, and IKKγ), exhibiting a particularly strong affinity for IKKα. FKBP51 depletion impaired IKKγ’s association with the catalytic subunits IKKα and IKKβ, suggesting FKBP51 serves as a structural scaffold essential for IKK kinase complex assembly (Romano et al., 2015). Beyond scaffolding, FKBP51 appears to act as a PPIase that modulates IKK activity. While the immunosuppressant FK506, an isomerase inhibitor, does not affect FKBP51/IKK binding or IKK complex formation, it inhibits IKK kinase activity (Jiang et al., 2008; Romano et al., 2015). In glioblastoma (GBM) U87 cells, FKBP51’s FK1 domain mediates cis-trans isomerization of peptidyl-prolyl bonds within IKK subunits (Jiang et al., 2008). Additional proteins in the NF-κB pathway, such as RelA, TRAF, and TBK1, have also been shown to interact with FKBP51 (Akiyama et al., 2014; Erlejman et al., 2014).

### 3.4 Tau-related mechanisms

Tau is a microtubule-associated protein predominantly expressed in neurons. It exists as multiple isoforms that play a crucial role in the assembly of tubulin monomers into microtubules and in maintaining the cytoskeleton and axonal transport. The abnormal hyperphosphorylation and aggregation of Tau into neurofibrillary tangles represent a hallmark feature of neurodegenerative diseases such as Alzheimer’s disease (Arnsten et al., 2025; O’Day, 2025).

Several studies have demonstrated that FKBP51 expression is strongly correlated with total tau protein levels. Decreased FKBP51 reduces tau protein accumulation, whereas elevated FKBP51 expression increases its levels (Blair et al., 2013; Jinwal et al., 2010; Oroz et al., 2018). Blair et al. (2013) revealed that FKBP51, in combination with HSP90, generates oligomeric tau in the brain, thereby enhancing its neurotoxic effects. Using small-angle X-ray scattering and nuclear magnetic resonance spectroscopy techniques, Oroz et al. (2018) reconstructed the tripartite HSP90/FKBP51/Tau complex. HSP90 acts as a scaffold, bringing FKBP51 and Tau proteins into proximity. FKBP51 then catalyzes the isomerization of proline residues in phosphorylated tau (p-tau) from trans to cis confirmations, accelerating Alzheimer’s disease (AD) progression (Oroz et al., 2018). Blair et al. (2013) also demonstrated that FKBP51 and HSP90 collaboratively impair tau degradation by inhibiting the 20S proteasome, leading to an accumulation of non-amyloid, neurotoxic tau oligomers. Furthermore, FKBP51 impairs tau ubiquitination, thereby shielding it from proteasomal degradation and contributing to its accumulation (Jinwal et al., 2010). Notably, the regulation of tau by FKBP51 is complex and involves a functional competition between its distinct activities. The PPIase activity of the FK1 domain may promote tau aggregation, whereas the chaperone function mediated by its TPR domain—particularly when forming complexes with co-chaperones like p23—can attenuate aggregation (Chakraborty and Zweckstetter, 2025). This functional competition appears to be concentration-dependent, with lower concentrations favoring PPIase activity, while higher concentrations may allow the chaperone activity to dominate (Chakraborty and Zweckstetter, 2025; Jinwal et al., 2010). Recent research further elucidates that p23 and FKBP51 can form a dynamic p23-FKBP51-tau ternary complex, which delays tau aggregation and may counteract the toxicity mediated by the HSP90-FKBP51 axis, providing a new molecular basis for understanding the dual role of FKBP51 in tau pathology (Chakraborty and Zweckstetter, 2025).

### 3.5 Other FKBP51-mediated pathways

Beyond the core pathways described above, FKBP51 is also extensively involved in the pathogenesis of central nervous system (CNS) diseases by regulating key processes such as macroautophagy, secretory autophagy, DNA methylation, and immune responses.

FKBP51 serves as a critical regulator of autophagy. Gassen et al. (2014) demonstrated that FKBP51, through its scaffolding function, enhances AKT activation, thereby inhibiting mTORC1 signaling and negatively regulating the initiation of macroautophagy. This mechanism is particularly relevant in protein-aggregation diseases such as Huntington’s disease, where impaired autophagy directly compromises the clearance of mutant proteins like mHTT (Wold et al., 2016). Furthermore, emerging evidence has revealed a role for FKBP51 in secretory autophagy. Martinelli et al. (2021) and Hartmann et al. (2024) discovered that FKBP51 interacts with core autophagy proteins such as ATG5, influencing the secretion of specific autophagy-related vesicles. This process may regulate the release of pathological factors, including cytokines and misfolded proteins, thereby playing a significant role in shaping the neuroinflammatory and tumor immune microenvironments.

At the epigenetic level, FKBP51 can influence the methylation status of genes. Gassen et al. (2015) confirmed that FKBP51 physically interacts with DNA methyltransferase 1 (DNMT1) and promotes DNA methylation in the promoter regions of its target genes. This regulatory mechanism has profound implications for the long-term programming of stress-responsive genes, linking early-life stress exposure to an increased susceptibility to subsequent neuropsychiatric disorders and cognitive dysfunction.

In terms of immunomodulation, in addition to the NF-κB pathway, FKBP51 may exert effects through other mechanisms. Early work by Li et al. (2002) suggested that FKBP51, potentially via its PPIase activity, may modulate the Nuclear Factor of Activated T-cells (NFAT) signaling pathway, thereby influencing T-cell activation and cytokine production. Although the specific functions of this pathway in the nervous system require further exploration, it offers a novel perspective for understanding the potential role of FKBP51 in neuro-immune interactions.

## 4 Role of FKBP51 in neurological diseases

### 4.1 Alzheimer’s disease

FKBP51 contributes to Alzheimer’s disease (AD) progression through its age-dependent upregulation, direct interactions with core pathological proteins, and modulation of stress-related signaling pathways (Blair et al., 2013; Jinwal et al., 2010; Roe et al., 2023). Studies suggest that elevated FKBP51 expression correlates with disease progression, supporting its potential as both a biomarker and a therapeutic target in AD (Blair et al., 2015; Roe et al., 2023). In experimental models combining amyloid-beta (Aβ) pathology and stress stimuli, persistent FKBP51 upregulation in the hippocampus and amygdala correlates with the severity of memory deficits (Faborode et al., 2022).

Mechanistically, FKBP51 interacts with key pathological components of AD. It colocalizes with hyperphosphorylated tau in neuronal microtubules and physically associates with Aβ oligomers (Faborode et al., 2022; Koren et al., 2011). Moreover, FKBP51 modulates GR signaling, potentially influencing tau and Aβ pathways through nuclear translocation regulation, though the precise mechanisms remain to be fully elucidated (Blair et al., 2015; Escribano et al., 2009; Wochnik et al., 2005). Emerging evidence also points to intricate molecular networks involving FKBP51, such as inverse correlations between Bin1 and NR2B expression and direct FKBP51-NR2B associations in AD-vulnerable brain areas (Faborode et al., 2022). These findings suggest that FKBP51 acts as a central mediator, integrating Aβ toxicity, tau dysfunction, and stress-related pathways. More details of FKBP51 in AD-related studies are presented in Table 1.

**TABLE 1:** FKBP51’s interactions with various molecular pathways and their potential implications in AD.

Molecules	Experimental drugs	Effects of FKBP51	Conclusion	References
FKBP51 tau	Not specified	FKBP51 forms a chaperone complex with HSP90, which prevents the degradation of tau protein, stabilizes tau and regulates its phosphorylation.	FKBP51 plays a significant role in tauopathies, particularly AD, by influencing tau protein’s stability, aggregation, and neurotoxicity. The increase in FKBP51 levels with age and in AD, potentially due to changes in DNA methylation.	Blair et al., 2013
FKBP51, tau, HSP90	LA1011	FKBP51 promotes the hyperphosphorylation of tau, preserving neurotoxic tau.	LA1011 hindered the attachment of FKBP51 to HSP90, leading to a decrease in tau pathology and the development of amyloid plaques.	Roe et al., 2023
FKBP51, tau, HSP90	Not specified	1. FKBP51 transforms tau into an isomer, influencing its engagement with the chaperone system and preparing it for dephosphorylation. 2. FKBP51 collaborates with the HSP90 complex in recycling tau to stabilize microtubules. 3. It seems that FKBP51 maintains tau protein by hindering its ubiquitination process.	The PPIase activity of FKBP51 is crucial in these processes, affecting tau’s phosphorylation state and its interactions within the cellular chaperone system.	Jinwal et al., 2010
FKBP51, Bin1, NR2B, Aβ	Not specified	A reverse relationship was observed between the duration spent in the Morris water maze’s target quadrant (a marker of improved memory) and the expression of FKBP51 in both the amygdala and hippocampus.	1. A rise in FKBP51 expression correlates with a decrease in memory. 2. Trauma-like stress and Aβ pathology each have the potential to independently and jointly affect the expression of FKBP51.	Faborode et al., 2022
FKBP51 HSP90	Not specified	FKBP51’s TPR domain facilitates its interaction with HSP90 through its C-terminal MEEVD peptide. The C-terminal peptide of HSP90 attaches to FKBP51’s TPR domain using a di-carboxylate clamp that includes Lys272, Glu273, Lys352, Asn322, and Lys329.	Initiatives to adjust the levels of FKBP51 or its interplay with HSP90 might hold therapeutic significance in treating Alzheimer’s disease, tauopathies, and disorders related to stress.	Kumar et al., 2017
FKBP51, tau, HSP90, p23	Not specified	1. *In vitro* experiments demonstrated that although FKBP51 alone did not alter the kinetics of tau aggregation, it significantly enhanced the fluorescence intensity of Thioflavin T (ThT), suggesting its potential to modify the architecture of tau fibrils or facilitate the formation of more densely packed amyloid fibers. 2. When forming a complex with the co-chaperone p23, FKBP51 participated in constituting a dynamic p23-FKBP51-tau ternary complex, which decelerated tau aggregation and potentially counteracted the toxicity mediated by the HSP90-FKBP51 axis.	FKBP51 plays a dual role in Alzheimer’s disease pathogenesis: on one hand, it stabilizes tau and facilitates its toxic oligomerization through an HSP90-dependent mechanism; conversely, when forming a complex with p23, it may exert a protective function via an HSP90-independent pathway.	Chakraborty and Zweckstetter, 2025

In conclusion, FKBP51’s multifaceted involvement in tau pathology and stress signaling highlights its significance in AD. Future research should prioritize three areas: (1) defining FKBP51’s temporal role throughout AD progression; (2) validating its biomarker potential through longitudinal clinical studies; and (3) elucidating the molecular crosstalk between FKBP51-mediated signaling and established AD therapeutic targets.

### 4.2 Parkinson’s disease

Parkinson’s disease (PD) is a neurodegenerative disorder characterized by motor and non-motor symptoms, primarily due to the loss of dopaminergic neurons in the substantia nigra (Kalia and Lang, 2015). Its etiology involves complex interactions among genetic, environmental, and age-related factors (Kalia and Lang, 2015; Wang et al., 2016). Among these, mutations in the PTEN-induced putative kinase 1 (PINK1) gene are linked to autosomal recessive early-onset PD (Wang et al., 2016). Recent studies have demonstrated a marked upregulation of FKBP51 in the brains of individuals with Parkinson’s disease (PD) (Garcia-Gomara et al., 2025).

Recent studies have implicated FKBP51 in PD pathogenesis, particularly via the PINK1 signaling axis (Boonying et al., 2019). In primary cortical neurons from Pink1-deficient mice, elevated FKBP51 levels reduce AKT phosphorylation at Ser473 following exposure to 1-methyl-4-phenylpyridinium (MPP^+^), a neurotoxin used to model PD (Boonying et al., 2019). This reduction increases neuronal susceptibility to cell death. Notably, in the presence of functional Pink1, FKBP51 is directly phosphorylated at a serine residue, which suppresses its inhibitory effect on AKT activation—thereby protecting neurons against MPP-induced neurotoxicity (Boonying et al., 2019). FKBP51 also interacts with the phosphatase PHLPP, contributing to AKT dephosphorylation and inactivation (Pei et al., 2009). In the absence of active PINK1, the FKBP51-PHLPP interaction is enhanced, leading to exacerbated dephosphorylation and inactivation of AKT, which in turn increases neuronal vulnerability (Boonying et al., 2019). Consequently, experimental inhibition of FKBP51 restores AKT signaling and ameliorates MPP^+^-induced cytotoxicity, confirming the pathogenic role of this enhanced interaction in PD models.

Collectively, these findings position the FKBP51-PINK1-AKT axis as a promising therapeutic target for PD. Future work is needed to fully elucidate the cell-type specific functions of FKBP51 across different stages of the disease.

Collectively, therapeutic strategies targeting disruption of FKBP51-PINK1 interactions may offer novel approaches for preserving dopaminergic neurons by maintaining physiological AKT signaling. Current findings suggest that regulating FKBP51’s phosphatase-binding capacity prevents pathological AKT inactivation in degenerating neurons. Mapping the complete network of FKBP51’s molecular partnerships within neuronal subtypes and investigating its context-dependent functions across disease stages are necessary. With the elucidation of these cell-type specific regulatory mechanisms, FKBP51-targeted interventions can be translated into clinically viable treatments while minimizing off-target consequences.

### 4.3 Huntington’s disease

Huntington’s disease (HD) is caused by autosomal dominant mutations in the HTT gene on chromosome 4, resulting in abnormally expanded CAG repeats that encode polyglutamine chains in huntingtin (HTT) proteins (The Huntington’s Disease Collaborative Research Group, 1993). These misfolded proteins accumulate and aggregate, disrupting neuronal function and viability. Current therapeutic efforts aim to selectively degrade mutant HTT while sparing normal HTT function.

Research has demonstrated a significant downregulation of FKBP51 expression across multiple Huntington’s disease (HD) models, including the striatum and cortex of R6/2 and zQ175 transgenic mice, as well as in human induced pluripotent stem cell (iPSC)-derived HD neural stem cells (NSCs) and medium spiny neurons (MSNs) (Bailus et al., 2021). This reduction in expression exhibits distinct regional specificity and temporal dependency, being detectable as early as the initial disease stages and progressively worsening with disease advancement. Co-immunoprecipitation and co-localization studies confirm a physical interaction between FKBP51 and the HTT protein, suggesting its potential involvement in HD pathogenesis by influencing the conformational state or aggregation propensity of HTT (Bailus et al., 2021). Notably, despite the observed decrease in FKBP51 levels in HD, further suppression of its activity via siRNA-mediated knockdown or pharmacological inhibition with the specific inhibitor SAFit2 significantly reduces mutant HTT (mHTT) protein levels, indicating a complex pathophysiological role for FKBP51 in HD.

FKBP5 regulates the degradation of HTT through direct physical interaction, thereby influencing the progression of Huntington’s disease (HD). Experimental suppression of FKBP51—genetically or pharmacologically—enhances the levels of autophagic markers LC3 and SQSTM1/p62 and reduces mHTT accumulation in HD NSCs and mouse models (Bailus et al., 2021). These findings suggest FKBP51 may negatively regulate the autophagic removal of toxic HTT species, and suggest a new avenue for therapeutic intervention (Wold et al., 2016). Notably, when FKBP51 is manipulated, it exerts bidirectional effects on the autophagic machinery, with its suppression improving LC3-mediated degradation pathways that reduce the toxic mHTT burden. This regulatory capacity presents a two-pronged therapeutic opportunity: disrupting pathological protein assemblies while amplifying intrinsic neuroprotective clearance systems. Experimental data unveiling FKBP51-mediated enhancement of autophagic markers have presented particularly promising implications given the impairment of protein degradation systems in HD progression (Wold et al., 2016).

In the future, longitudinal assessments of FKBP51-targeted interventions must be conducted, particularly while investigating their disease-modifying potential across HD progression stages in translational models. Cell-type dependent regulatory mechanisms must be studied to further understand the regional specificity of FKBP51 depletion in striatal neurons. Additional mechanistic studies are warranted to fully delineate the therapeutic window of FKBP51 and potential off-target effects within neuronal populations susceptible to HD pathology.

### 4.4 Ischemic stroke

Ischemic stroke is an acute cerebrovascular disease characterized by a disruption of the blood supply to the brain, leading to ischemic and hypoxic necrosis of brain tissue, which consequently results in the sudden onset of neurological deficits (Lindsay et al., 2019). Recently, growing attention has focused on the role of FKBP51 in ischemic stroke and its associated pathological mechanisms (Liu et al., 2022; Wegner et al., 2019; Yu et al., 2019, 2020).

FKBP51 is a key determinant of stroke outcome by regulating neuronal survival and endothelial cell proliferation. Following ischemic stroke, FKBP51 is upregulated in response to cerebral ischemia, indicating its involvement in the post-stroke cellular response (Table 2; Liu et al., 2022; Wegner et al., 2019; Yu et al., 2020). This upregulation has been shown to aggravate neuronal injury by enhancing inflammation and autophagy (Yu et al., 2020). Specifically, FKBP51 overexpression activates autophagy and reduces neuronal viability following oxygen-glucose deprivation/reoxygenation (OGD/R) injury, mediated by the AKT/FoxO3-dependent pathway (Yu et al., 2020). The increased expression of miR-214-3p boosted cell proliferation, angiogenesis, migration, and hindered apoptosis in human brain microvascular endothelial cells under OGD conditions, FKBP51 overexpression attenuates these beneficial effects (Liu et al., 2022). Furthermore, given the established role of FKBP51 in NF-κB pathway-mediated immune inflammation, its increased expression can amplify inflammation and cardiovascular risk by activating the master immune regulator NF-κB, thereby exacerbating the pro-inflammatory environment in ischemic stroke (Zannas et al., 2019). Notably, increased FKBP51 mRNA levels positively correlate with infarct volume in patients with acute ischemic stroke, suggesting its potential as a prognostic biomarker. A key priority for future work is to define the time-specific mechanisms of FKBP51 in stroke. This requires a combined approach of pharmacological targeting with selective inhibitors in MCAO models, and a longitudinal assessment of FKBP51 expression and neuroimaging biomarkers in patient cohorts.

**TABLE 2:** Changes in FKBP51 expression after stroke.

Type of experiment	Subject	Treatment	Results	References
Animal experiment	Male 129S6/SvEv mice	Chronically stress and stroke	Levels of FKBP51 mRNA and proteins rise in both the hypothalamus and the ischemic brain tissue of mice.	Wegner et al., 2019
Animal experiment	Male C57BL/6J mice	Transient middle cerebral artery occlusion (tMCAO)	The expression of FKBP51 is upregulated in both plasma and brain tissues after tMCAO.	Yu et al., 2020
Cell experiment	Human brain microvascular endothelial cells (HBMECs)	Oxygen-glucose deprivation (OGD)	In OGD-treated HBMECs, FKBP51 levels were increased	Liu et al., 2022
Cell experiment	Neuro-2a cells	oxygen and glucose deprivation and reoxygenation (OGD/R)	Both mRNA and protein levels of FKBP51 increased notably after OGD/R treatment.	Yu et al., 2020
Clinical study	Plasma samples from participants	AIS patients received thrombolytic therapy	FKBP51 was upregulated significantly in AIS patients.	Yu et al., 2020

### 4.5 Glioma

A growing body of literature has investigated FKBP51’s role in glioma pathophysiology (Table 3). FKBP51 contributes to multiple processes, including tumor growth, invasiveness, immune evasion, and treatment resistance.

**TABLE 3:** FKBP51’s interactions with various molecular pathways and their potential implications in glioma.

Molecules	Drugs	Mechanisms	Conclusions	References
FKBP51 and PD-L1	Celecoxib	In both the glioma model and human GBM cells grown in culture, celecoxib was found to decrease PD-L1 levels. The decrease was associated with the post-transcriptional control of FKBP51.	Combining celecoxib with anti-PD-1 antibody therapy offers promising antitumor effects in GBM by targeting PD-L1 expression.	Yamaguchi et al., 2020
FKBP51 and AKT	Not specified	Elevated levels of FKBP51 led to a decrease in AKT phosphorylation at Ser473, a reduction in the anti-apoptotic protein Bcl-2, an increase in the pro-apoptotic protein Bax, and a rise in the splitting of caspase-9 and caspase-3.	In glioma, FKBP51 functions as a tumor inhibitor, blocking AKT activation and activating the inherent mitochondrial apoptotic route.	Yang et al., 2015
FKBP51 and NF-κB	Rapamycin	Elevated levels of FKBP51 in glioma cells correlated with enhanced cell proliferation, potentially linked to FKBP51’s role in triggering the NF-κB pathway.	FKBP51 significantly influences glioma development and resistance to chemotherapy through the modulation of the NF-κB signaling route. Targeting FKBP51 could enhance the effectiveness of rapamycin treatment in glioma, providing a potential therapeutic strategy.	Jiang et al., 2008
FKBP51	Carmustine	FKBP51 is highly expressed in BT325 cells, which induces cell cycle arrest and reduces tumor cell proliferation by inhibiting the phosphorylation of AKT ser473 and activating the transcription of P21 and P27. At the same time, the overexpression of FKBP51 can increase the sensitivity of BT325 to BCNU through the AKT pathway. On the other hand, FKBP51 can increase the expression of MMP-2 and MMP-9 in BT325 cells and enhance the migration and invasion ability of cells.	FKBP51 has complex effects on BT325 glioma cells: inhibiting proliferation and sensitizing cells to BCNU, while promoting migration and invasion. The AKT and NF-κB pathways facilitate these impacts. FKBP51 may act as a predictive biomarker for glioma and a marker for how patients react to chemotherapy.	Li et al., 2020
FKBP51S and PD-L1	SAFits	FKBP51s upregulates PD-L1 expression via protein folding and glycosylation, which protects the tumor from its immune microenvironment.	SAFits, a selective inhibitor of KBP51, reduced PD-L1 expression and PD-L1-induced cell death in coculture of peripheral blood mononuclear cells with glioma cells. This indicates a potential immunomodulatory strategy for treating glioblastoma	D’Arrigo et al., 2017
FKBP51s and PD-L1	SAFit2	FKBP51 promotes GBM malignancy and stemness, influences PD-L1 expression	Targeting FKBP51s can effectively reduce PD-L1 expression and hinder GBM malignancy, indicating a potential therapeutic strategy for treating GBM.	D’Arrigo et al., 2019
FKBP51S, cyclin D and PD-L1	Not specified	PD-L1 expression rises with cyclin D during G1/S transition, decreasing in exponential cell growth. FKBP51s localizes to the endoplasmic reticulum during cyclin D and PD-L1 peaks, shifting to the nucleus during cell division. FKBP51s engages with the CCND1 promoter within enclosed chromatin, indicating its function in controlling cyclin D expression.	PD-L1 expression in GBM cells is dynamically controlled, correlating with cell proliferation and cyclin D levels. FKBP51s, a co-chaperone of PD-L1, is implicated in these processes, indicating its potential role in cyclic cyclin D expression during the cell cycle.	Tufano et al., 2021
FKBP51	Not specified	FKBP51 is preferentially expressed in macrophages and microglia in human glioblastoma.	FKBP51 could be crucial in determining cell destiny, movement, tumor development, tumor advancement, and the formation of new blood vessels.	Rotoli et al., 2019

The role of FKBP51 in glioma growth appears to be context-dependent, exhibiting both pro- and anti-tumorigenic effects. For instance, D’Arrigo et al. (2019) reported that FKBP51s, a spliced isoform lacking the TPR domain, enhances the anti-apoptotic capacity of GBM cells, particularly those with cancer stem-like features. Conversely, Yang et al. (2015) demonstrated that the full-length FKBP51 (isoform 1) suppresses glioma growth by increasing Bcl-2 expression and decreasing Bax, and cleaved caspase-3 and caspase-9, thereby hindering cell growth and promoting apoptosis. This tumor-suppressive effect is mediated through the inhibition of the AKT pathway and activation of the mitochondrial apoptosis pathway. Similarly, Li et al. (2020) showed that the full-length FKBP51 (isoform 1) inhibits glioma cell proliferation, reduces glioma cell viability, and enhances sensitivity to the chemotherapeutic agent carmustine (BCNU). This evidence indicates that FKBP51 plays a dual role in inhibiting and promoting tumor properties. These seemingly contradictory findings may be partly attributed to the specific FKBP51 isoforms examined in different studies. Crucially, the full-length isoform (FKBP51, i.e., isoform 1) and the shorter splice variant (FKBP51s, i.e., isoform 2) exhibit key structural and functional differences: FKBP51s lacks the C-terminal TPR domain, which is critical for interactions with chaperones like HSP90, and possesses a short unique sequence at its C-terminus (Martinelli et al., 2024). This structural divergence likely underlies their opposing or distinct roles in regulating different signaling pathways. Furthermore, glioma cell line-specific differences in FKBP51 regulation or variability in *in vitro* conditions may also contribute to the observed discrepancies, warranting further investigation.

FKBP51 also supports glioma cell migration and invasion. Li et al. (2020) reported that FKBP51 upregulates phosphorylated IKKα (p-IKKα), and matrix metalloproteinases MMP-2 and MMP-9, which are critical mediators of tumor invasiveness.

Furthermore, FKBP51 controls how gliomas evade immune detection. In gliomas, FKBP51 and its isoform FKBP51s are key regulators of the expression of programmed death ligand 1 (PD-L1), a protein essential for tumor immune evasion. D’Arrigo et al. (2017) and Tufano et al. (2021) showed that FKBP51s enhance PD-L1 surface expression via protein folding and glycosylation processes. This regulation is critical because PD-L1 is pivotal for reducing the host’s antitumor immunity. Interestingly, Yamaguchi et al. (2020) found that celecoxib downregulates PD-L1 in glioma cells by modulating FKBP51 post-transcriptionally. Tufano et al. (2021) further suggested that FKBP51s may coordinate PD-L1 expression with cell cycle progression via cyclin D, suggesting a complex regulatory axis that deserves deeper exploration.

FKBP51 is also implicated in improving treatment resistance in patients. D’Arrigo et al. (2019) emphasized FKBP51’s role in enhancing GBM aggressiveness and resistance to therapy. By conducting a random sampling of glioma tumors, Jiang et al. (2008) reported a positive correlation between FKBP51 expression and glioma grade, with higher FKBP51 levels associated with poorer patient survival (Jiang et al., 2008). Therefore, targeting FKBP51 may improve the malignancy or drug resistance of gliomas. For example, FKBP51 inhibition increases glioma sensitivity to BCNU (Li et al., 2020) and FKBP51 knockout enhances responsiveness to rapamycin (Jiang et al., 2008), suggesting therapeutic potential in targeting FKBP51 to overcome resistance.

In conclusion, FKBP51 plays a dual role in glioma dynamics: its spliced isoform, FKBP51s, promotes malignancy by enhancing anti-apoptotic capacity in GBM stem-like cells, whereas the full-length FKBP51 (isoform 1) can inhibit tumor growth by suppressing proliferation and inducing apoptosis through AKT pathway modulation. In immune evasion, FKBP51s is a key regulator of PD-L1 expression, thereby modulating antitumor immunity. Moreover, FKBP51 contributes to treatment resistance, with FKBP51s augmenting GBM malignancy and influencing therapeutic responsiveness. Furthermore, the link between FKBP51 expression levels and glioma severity, combined with the structural and functional distinctions between its isoforms (FKBP51 isoform 1 vs. FKBP51s/isoform 2), highlights its potential as a therapeutic target to improve treatment efficacy.

## 5 Current therapeutic strategies targeting FKBP51 in neurological diseases

### 5.1 Traditional medicine

Rapamycin, widely known for its immunosuppressive properties, has gained prominence in neuroscience for its ability to inhibit the mTOR-signaling pathway, which is pivotal for synaptic plasticity, neuronal survival, and neurogenesis (Ding et al., 2021; Sarkar et al., 2008). Rapamycin acts by binding to FKBP12, a close homolog of FKBP51, thereby inhibiting downstream mTOR signaling (Jiang et al., 2008). Despite the rapamycin-FKBP12 complex does not directly interact with FKBP51, the interconnected nature of cellular signaling suggests that mTORC1 inhibition could indirectly influence FKBP51’s function. Studies have indicated that FKBP51 influences cell proliferation and responsiveness to rapamycin by modulating NF-κB phosphorylation, thereby playing a key role in regulating glioma growth and chemoresistance through the NF-κB pathway (Jiang et al., 2008).

Celecoxib, an NSAID traditionally recognized for COX-2 inhibition, has demonstrated anticancer properties in various malignancies (Tołoczko-Iwaniuk et al., 2019; Yamaguchi et al., 2020; Zhang et al., 2019). Notably, it modulates FKBP51 expression in glioma cells, leading to reduced PD-L1 levels and tumor aggressiveness. Such modulation may disrupt tumor immune evasion mechanisms and enhance sensitivity to anti-PD-1 immunotherapy (Yamaguchi et al., 2020). These findings expand our understanding of celecoxib’s antitumor mechanisms and establish FKBP51 as a critical regulator of cancer immunomodulation. The identified FKBP51-PD-L1 regulatory axis emerges as a strategic target for combinatory therapies, particularly in treatment-resistant gliomas. Current investigations position FKBP51 manipulation as promising for amplifying immune checkpoint blockade efficacy, thus highlighting its emerging significance as a therapeutic target in immuno-oncology. Further investigation into celecoxib’s FKBP51-mediated pathways could improve its clinical application while mitigating traditional NSAID-associated adverse effects.

### 5.2 Small molecule inhibitors-SAFits

SAFits, particularly SAFit1 and SAFit2, represent a class of small molecule inhibitors that selectively target FKBP51, offering novel therapeutic potential in neurological and oncological disorders. They exhibit disease-modifying effects in HD and GBM through distinct molecular mechanisms (Bailus et al., 2021; D’Arrigo et al., 2017, 2019; Marrone et al., 2025).

In HD pathology, SAFit2 disrupts the FKBP51–mHTT interaction, directly reducing toxic protein accumulation. In experimental models, SAFit2 augments autophagic clearance of mHTT independent of mTOR signaling. This distinguishes its mechanism from those of conventional therapies like rapamycin (Bailus et al., 2021). This strategy addresses a major challenge in managing protein aggregation in neurodegenerative disorders.

In GBM, SAFits target the FKBP51s isoform, which facilitates immune evasion by regulating PD-L1 maturation. These compounds reduce tumor immunosuppression and amplify apoptosis (D’Arrigo et al., 2017, 2019). This dual mechanism—suppressing immune checkpoint molecules and promoting cell death—positions SAFits as valuable adjuvants to current immunotherapies in glioma treatment (D’Arrigo et al., 2017, 2019). The isoform-specific action of SAFits highlights the potential of precision-targeted therapeutics. Their utility may extend beyond HD and GBM to diverse FKBP51-associated conditions, including ischemic stroke and AD. Future efforts should focus on optimizing SAFit selectivity and specificity, minimizing off-target effects, and validating their clinical relevance across disease models.

However, it is noteworthy that the efficacy of SAFit can be context-dependent. A recent study by Marrone et al. (2025) demonstrated that while SAFit treatment effectively suppressed intrinsic tumor survival pathways such as NF-κB and TGF-β signaling in melanoma cells *in vitro*, it concurrently induced an immunosuppressive tumor microenvironment characterized by M2 macrophage polarization and CD8+ T-cell exhaustion in a syngeneic mouse model, which ultimately compromised its *in vivo* efficacy. This highlights the complex, and sometimes opposing, roles of FKBP51 inhibition in different pathological contexts, underscoring the importance of thoroughly evaluating the tumor immune microenvironment when developing FKBP51-targeted therapies, especially for cancers.

### 5.3 Gene editing technologies

Gene-silencing strategies targeting FKBP51—such as siRNA—demonstrate significant promise in neurodegenerative and oncological models. In HD, suppressing FKBP51 expression reduces mHTT accumulation and promotes neuroprotection (Bailus et al., 2021). In glioma, siRNA-mediated FKBP51 knockdown alters cancer cell proliferation and enhances apoptosis, highlighting its tumor-suppressive effects (D’Arrigo et al., 2017, 2019; Pei et al., 2009; Yang et al., 2015). These findings catalyze interest in advanced gene-editing approaches. CRISPR/Cas9 systems offer a more durable gene-editing approach, potentially enabling durable FKBP51 regulation through genomic modifications (Zhou et al., 2019). In neurodegenerative diseases like HD, permanent FKBP51 editing disrupts pathogenic protein interactions. In GBM, sustained FKBP51 modulation might increase chemosensitivity by disrupting AKT pathway dynamics. However, transitioning from transient silencing to permanent editing raises several challenges. Delivery systems must be optimized for tissue-specific targeting with minimal off-target effects. Additionally, because FKBP51 participates in diverse regulatory networks, comprehensive functional mapping is required to prevent unintended adverse consequences. Ethical concerns, particularly regarding permanent genomic alterations, must also be addressed (Hough and Ajetunmobi, 2017). Furthermore, current priorities include developing isoform-specific editing strategies capable of distinguishing between FKBP51 isoforms and validating their therapeutic windows in preclinical models (Martinelli et al., 2024). This approach is particularly crucial given the recent findings that FKBP51 isoforms exhibit distinct expression dynamics, subcellular localization, and functional roles in key pathways such as glucocorticoid receptor signaling, autophagy, and immune regulation, underscoring the need for isoform-selective therapeutic interventions (Martinelli et al., 2024).

## 6 Conclusion

A growing body of evidence solidifies FKBP51 as a central scaffolding protein and pleiotropic signaling hub in the pathophysiology of a wide spectrum of central nervous system disorders. By integrating key pathways—including the HSP90 chaperone system, glucocorticoid receptor signaling, AKT, and NF-κB—FKBP51 exerts critical regulatory functions in neurodegenerative diseases, cerebrovascular disorders, and intracranial tumors. In Alzheimer’s disease, the FKBP51- HSP90 complex drives the conformational conversion and oligomerization of tau protein. In models of ischemic stroke, its upregulation exacerbates neuronal injury via the AKT/FOXO3 pathway. Most notably, in glioma, FKBP51 exhibits a unique context-dependent duality, potentially suppressing tumor growth via AKT inhibition while simultaneously promoting immune escape through regulation of PD-L1 expression.

These mechanistic insights provide a compelling rationale for therapeutic strategies targeting FKBP51. Preclinical studies have demonstrated the potential of selective small-molecule inhibitors like SAFit2 to ameliorate disease phenotypes, while gene-editing technologies offer novel avenues for long-term intervention. However, the translation of these findings faces several interconnected challenges. Future research must first deepen our understanding of FKBP51’s cell-type and disease-stage specific functions, and decipher the context-dependent networks that dictate its dual roles. Concurrently, efforts should advance the optimization and clinical validation of highly selective inhibitors and isoform-specific tools, which are crucial for dissecting its complex biology and minimizing off-target effects. Exploring rational combination therapies that leverage FKBP51 inhibition alongside existing modalities represents another promising frontier. Finally, the biomarker potential of FKBP51 requires rigorous validation in large, longitudinal clinical cohorts to assess its utility in diagnosis, prognosis, and treatment stratification.

In summary, as a critical node converging multiple pathogenic pathways, FKBP51 represents a prime target for therapeutic intervention. The paramount challenge for the field lies in unraveling the determinants of its functional duality and harnessing this knowledge to propel targeted strategies toward successful clinical translation.
